# The Impact of Rural Alimentation on the Motivation and Retention of Indigenous Community Health Workers in India: A Qualitative Study

**DOI:** 10.2196/48346

**Published:** 2025-01-23

**Authors:** Ajit Kerketta, Raghavendra A N

**Affiliations:** 1 CHRIST (Deemed to be University), Hosur Road, Bhavani Nagar, Bengaluru, 560029, India, 91 8867055238

**Keywords:** rural alimentation, community health workers, motivation, retention, rural health, rural nutrition, workforce

## Abstract

**Background:**

Rural health care delivery remains a global challenge and India is no exception, particularly in regions with Indigenous populations such as the state of Jharkhand. The Community Health Centres in Jharkhand, India, are staffed by Indigenous workers who play a crucial role in bridging the health care gap. However, their motivation and retention in these challenging areas are often influenced by a complex mix of sociocultural and environmental factors. One such significant but understudied influencing factor is alimentation, or nutrition, in rural settings. Previous studies have identified several motivators, including community ties, cultural alignment, job satisfaction, and financial incentives. However, the role of alimentation in their motivation and retention in rural areas has not been sufficiently explored.

**Objective:**

This study aims to explore how the strong bond with locally produced food products impacts the retention of Indigenous community health workers (CHWs) in Jharkhand, India, and shed light on a crucial aspect of rural health care workforce sustainability.

**Methods:**

This study adopted a phenomenological research design to explore the lived experiences and perspectives of Indigenous CHWs in Jharkhand. A purposive sampling method was used to select CHWs who had worked in rural areas for at least five years. Data were collected through semistructured interviews, focusing on the participants’ experiences of rural alimentation and how it influences their motivation and retention for rural health care. The interviews were audio recorded, transcribed, and analyzed using thematic analysis to identify common themes and patterns in their experiences related to nutrition and retention.

**Results:**

The study revealed that rural alimentation plays a significant role in both the motivation and retention of CHWs in Jharkhand. CHWs who experienced consistent access to local food reported higher job satisfaction, better physical well-being, and a stronger commitment to their roles. It has also been perceived that consuming nutrient-dense food products decreases the risk of chronic illness among rural populations. Additionally, community support systems related to alimentation were found to be crucial in maintaining motivation, with many CHWs emphasizing the importance of local food availability and cultural ties. The findings suggest that improving access to organic nutrition can positively influence the retention of CHWs in rural areas.

**Conclusions:**

Indigenous communities have unique food habits and preferences deeply rooted in agriculture and arboriculture. Their traditional eating practices are integral to their rich cultural heritage, with significant social, symbolic, and spiritual importance. This study highlights the critical role of rural alimentation in motivating and retaining CHWs in rural Community Health Centres in Jharkhand. Therefore, addressing organic versus conventional food in rural health care policies plays a vital role in improving the retention rates of CHWs. By recognizing the interconnectedness of nutrition and workforce sustainability, health care systems can better support Indigenous CHWs and continue delivering health care services.

## Introduction

### Rural Health and Alimentation

Community health workers (CHWs) play a vital role in providing primary health care services to rural populations in low- and middle-income countries [1,2]. However, retaining them in rural areas is challenging, largely due to low motivation. One potential factor influencing their motivation and retention is access to a diverse and nutritious diet or rural alimentation [3]. Although the term “alimentation” has existed in the English language since the late 16th century, it is rarely used. In Latin-based languages like French, “alimentation” conveys a holistic view of how humans produce, procure, prepare, share, consume, and digest their food, encompassing human, technological, sociocultural, and environmental aspects [4].

### Significance of Alimentation in Rural Health Systems

The term “rural alimentation” in this study refers to the food that Indigenous people produce, acquire, prepare, share, consume, and digest; it is intimately linked to their sociocultural and environmental surroundings. Indigenous CHWs are also among those who are devoted to these tastes and preferences and will find it difficult to give up their native cuisine. Rural food products that are fresh, pure, unadulterated, nutrient dense, and low in pesticides appeal to CHWs [5]. This experience has lured CHWs to continue serving in rural health centers in Jharkhand, India. However, the lack of local and traditional food in metropolitan cities has negatively impacted their motivation, causing many health workers to select rural health care jobs [6]. Previous studies indicate that the desire for nutritious food in the urban setting significantly affects their motivation, job satisfaction, and retention rates. For example, a study in Ethiopia showed that providing nutritious food to CHWs increased job satisfaction and reduced attrition rates [7]. Similarly, a study conducted in Malawi demonstrated that CHWs accessing local food products were less likely to leave their jobs [8]. Despite the potential impact of rural alimentation on CHWs’ motivation and retention, there is limited research on this topic in Jharkhand. Therefore, the study seeks to explore the question: “How does access to diverse and organic food in rural Jharkhand influence the motivation and retention of Indigenous community health workers?”

The study also aims to explore how Indigenous CHWs in Jharkhand perceive the impact of rural alimentation on their motivation and retention. Investigating the connection between rural alimentation and the motivation and retention of Indigenous CHWs will provide valuable insights into the factors that influence their engagement and commitment. We also intend to share the findings with policy makers and health care stakeholders, invoking the implementation of policies that support the well-being of CHWs, promote local food, and attract adequate CHWs to rural Jharkhand.

### Study Background

Jharkhand is an eastern Indian state with a population of 39 million spread across 79,714 square kilometers (2019 census). Out of the total population, 24.05% reside in urban areas, while 75.95% live in rural areas [9]. Agriculture and agroforestry products are the primary sources of livelihood. However, the younger generation is increasingly migrating to metropolitan cities in search of better, more sustainable living opportunities. Most state regions are characterized by hills, rugged terrain, lakes, and rivers, presenting significant challenges. While some areas have plains and level topography nestled within natural surroundings, socioeconomic difficulties and a lack of infrastructure make it challenging for CHWs to stay in these locations. Consequently, Jharkhand faces a severe shortage of health workforce [10,11]. Approximately 80% of health care workers are stationed in metropolitan cities catering to the 24.05% of the population residing in urban areas, while 20% health care workers serve the 75.95% population living in rural areas [11-13]. Additionally, the population’s strong beliefs in spirit worship and reliance on local quacks and tantric practices for their ill health further contribute to the short supply of CHWs. This study aims to provide evidence-based insights into the factors that promote the retention of CHWs in rural areas.

## Methods

### Study Design

We used a qualitative case research design to help understand the perspectives, emotions, and behaviors of Indigenous CHWs and uncover their in-depth experiences [14]. This approach focuses on understanding the subjective meaning that drove CHWs to work in rural Jharkhand [15,16]. This study selected participants with 5 years of service records in the respective Community Health Centers (CHCs).

### Ethical Considerations

The study is not a clinical trial and, therefore, does not require registration to establish safety and efficacy standards. Nevertheless, ethical approval was obtained from the Institutional Review Board of CHRIST (Deemed to be University), Bangalore, India (CU: RCEC/00371/11/22). Written informed consent was obtained from each participant before conducting the interview. To further ensure the privacy of the participants, all names were changed to pseudonyms during the transcriptions of the text. However, the interviewers (AK and RNA) know the actual names of the interview participants. Each participant received a fixed remuneration of US $5.75 after completion of the interview as an acknowledgment of their time and contribution.

### Setting and Sample

The corresponding author randomly selected and visited 3 CHCs in the eastern districts of Jharkhand to pilot the survey. This visit played a key role in shaping the development of the research objective: to explore the impact of rural alimentation (local and traditional food systems) on the motivation and retention of Indigenous CHWs in rural India, as well as to establish a suitable research framework. CHWs from these randomly selected CHCs participated by completing a self-validated questionnaire with open-ended questions. In the main study, 30 CHWs were selected; of these, 10, 12, and 8 CHWs from the respective CHCs met the study criteria. They had served more than five years, expressed willingness to continue residing in rural areas, and were government employees. The study adopted a purposive sampling technique, which helped obtain rich, detailed, and relevant data that influenced the motivation and retention of CHWs in Jharkhand [17]. The male and female respondents were selected irrespective of their rural and urban backgrounds.

### Process of Data Generation

A total of 14 participants (4 male individuals and 10 female individuals) ultimately consented to participate in the interviews. However, 16 individuals declined, with some initially agreeing but later withdrawing due to hesitation from the novelty of such an interview process and discomfort with having their comments audio recorded. The participants were aged 30-60 years and expressed their desire to participate in individual, face-to-face or telephone interviews within 10 months. A follow-up interview was done after 4 and 6 months. Within 4 months, 8 interviews were conducted at the CHCs and 6 interviews were conducted in the home district of the reviewer [18,19]. Multimedia Appendix 1 shows the interview guidelines and questionnaire.

The round-1 interview was precise and relevant to the objectives mentioned above (in the *Setting and Sample* section) and, hence, did not require a reinterview of any participants. Interviews were conducted both face-to-face and remotely in Hindi, a language in which the authors are fluent and experienced in conducting qualitative case research. While consent was sought to audio record the interviews, many participants expressed unwillingness; as a result, the researchers took detailed notes instead.

The data were collected through individual, semistructured qualitative case research, with in-depth interviews conducted according to the established protocol matrix [20]. Questions regarding all main areas were posed, albeit in varying order. The interviews in the 4-month follow-up ranged between 6 and 37 minutes (average of 10 min), and interviews in the 6-month follow-up ranged between 5 and 13 minutes (average of 7 min).

### Research Team and Reflexibility

AK (research scholar in human resource management, male, aged 40 y) and RAN (PhD in human resource management, male, aged 48 y) solely conducted the interviews. After the interviews, the corresponding author listened to the audio recordings, with several breaks between every audio recording, and transcribed them.

### Analysis

We employed the general data analysis methodologies indicated below in the context of thematic analysis and read the texts multiple times to familiarize and better understand them [21]. Descriptive codes were then applied to data segments [22] relevant to the research question: how do the local food habits influence motivation and continuation of work, and do these factors impact decisions to remain in rural areas? This question was aligned with the objective of the study [23]. The coded data were grouped into themes using QDA Miner Lite software (Provalis Research), demonstrating the relationships between them and identifying themes using inductive methods. The themes were assessed and modified depending on their relevance to the data and the research topic, and they were blended as appropriate. After the themes were developed, they were further defined and given titles that accurately expressed their meanings [24]. Then, the researcher drafted the report. The thematic analysis involves a recursive process of moving back and forth between the data and the emerging themes. It is an iterative and reflexive process, requiring the researcher to consider their biases and assumptions throughout the analysis.

- In-depth investigation: This method provided an in-depth understanding of the study’s objectives and phenomena [25]. It enabled the researchers to collect data from multiple sources and examine them comprehensively.
- Contextual analysis: The qualitative case research design allowed the researchers to focus on the social, cultural, economic, and political factors influencing the phenomenon [26].
- Interpretive analysis: This approach involved identifying themes and interpreting them in the context of the research objectives [21,27].
- Flexible design: The qualitative case research design is adaptable, allowing the researchers to evolve the design as data are collected and analyzed [28]. To explore complex and context-specific issues in real-life settings, the interview provided comprehensive insights into the CHWs’ experiences, opinions, and perspectives regarding rural alimentation and its impact on their motivation and retention.

## Results

### Study Participants

We contacted 4 CHCs; however, the medical officer at one center declined to grant permission, citing concerns that the study might inadvertently violate government protocols. A total of 64 CHWs were contacted across the remaining 3 CHCs, who were directly appointed by the government and were under the age of 60 years. Table 1 shows that the majority of health workers were female, accounting for 52% (13/25), 61% (11/18), and 57% (12/21) across the 3 CHCs. Among the 14 participants, 71% (n=10) were female and 29% (n=4) were male. This sex disparity could be a potential area for further research, exploring why fewer male CHWs tend to remain in rural locations.

**Table 1.** Participants characteristics from CHCs^a^ A, B, and C. This table combines the demographic and workplace preferences of health care workers across the 3 centers (A, B, and C).

Characteristics	Center A (n=25), n (%)	Center B (n=18), n (%)	Center (n=21), n (%)
**Sex**			
Male	12 (48)	7 (39)	9 (43)
Female	13 (52)	11 (61)	12 (57)
**Age group (years)**			
≤30	3 (12)	5 (28)	4 (19)
≥30	22 (88)	13 (72)	17 (81)
**Residence**			
Rural origin	25 (100)	18 (100)	21 (100)
Urban origin	0 (0)	0 (0)	0 (0)
**Preferred workplace**			
**Rural area**	8 (32)	12 (67)	15 (71)
Male	2 (25)^b^	4 (33)^b^	4 (27)^b^
Female	6 (75)^b^	9 (67)^b^	11 (73)^b^
**Urban area**	17 (68)	6 (33)	6 (29)
Male	3 (18)^c^	1 (17)^c^	2 (33)^c^
Female	14 (82)^c^	5 (83)^c^	4 (67)^c^

a CHC: Community Health Centre.

b Percentages are based the number of workers who preferred a rural workplace as the denominator.

c Percentages are based the number of workers who preferred an urban workplace as the denominator.

Data were analyzed by constructing a thematic analysis, identifying patterns and themes as guided by the research questions and objectives [24,29]. Emerging themes were verified through member checking to ensure accuracy and validity. This study offers a comprehensive understanding and valid representations [30] of the perspectives and experiences of CHWs staying in rural Jharkhand. The focus is on a specific area within the CHCs, which is predominantly tribal dominated. The analysis identified themes that offered insights into the barriers and facilitators affecting CHWs’ access to and consumption of diverse and nutritious food, as well as how their food habits intersect with their roles as health promoters and caregivers.

The study explored three major themes, presented as main themes and their corresponding minor themes, as illustrated below. These themes reflect the perspectives, experiences, and perceptions of the Indigenous CHWs regarding their reasons for remaining in rural Jharkhand.

1. The impact of rural alimentation on Indigenous CHWs’ motivation
2. Retention trends among Indigenous CHWs
3. Correlations between nutritional support and job satisfaction

### Impact of Rural Alimentation on Indigenous CHWs’ Motivation

#### Health and Nutrition

Local food, often known as “field to plate,” plays a vital role in connecting Indigenous CHWs to rural health centers. Free from preservatives, pesticides, additives, and flavorings, this food comes straight from the field, offering freshness and abundance, which enhances both its quality and appeal.

Whenever people call me to see patients or visit their house, they offer me fresh produce from their farm and sometimes even “desi” (country) chicken for free. Where can you get such nutritious and healthy food in cities?

[Nurse BY, 4-month interview]

#### Community Engagement

A unique characteristic of Indigenous communities is their emphasis on communitarian living, characterized by strong bonds of sharing and caring for one another [31,32]. Farming serves as both a livelihood and a means of fostering community engagement and identity. Their connection to the land, local markets, and cultural festivals centered around regional cuisine strengthens their sense of belonging and deepens social ties within the community.

I visit the villages whenever I have time. During these visits, many people gather to sit and discuss the health and well-being of the community, and we motivate the children. On holidays and Sundays, I often take the village youth to the rivers for fishing.

[Doctor BA, 4-month interview]

#### Work-Life Balance

The concern among these CHWs is their inability to manage their domestic chores, as distance limits regular visits to the family and family affairs. The opportunity to serve in their home town facilitates work-life balance and positively impacts their physical and mental health, reducing stress, increasing job satisfaction, and enhancing productivity [33].

#### Cultural Connection

Food habits often represent a deep cultural bond and sense of belonging [34]. It makes them feel a strong connection to their heritage and traditions through the food they grew up with, making it more appealing to remain in their hometown.

We gather together and prepare meals for every celebration in common for all young and old.

[Accredited social health activist PK, 4-month interview]

### Retention Trends Among Indigenous CHWs

#### Recognition

In rural areas, doctors often receive deep respect and appreciation from the rural community. This sense of being valued and recognized enforced emotional fulfillment, encouraging CHWs to continue serving in these regions.

I feel like a celebrity, as wherever I go—whether it’s the market, the community, or my workplace—people honour and respect me immensely.

[Doctor DM, 4-month interview]

#### Career Intentions

The state government implemented various strategies to encourage medical students to serve in rural areas, including career growth incentives such as district quotas for entrance into Bachelor of Medicine, Bachelor of Surgery programs; specialized training programs (eg, barefoot doctor training) for rural service; a 3-month community medicine internship in rural settings; government-sponsored quotas for postgraduate, diploma, and degree course selections; as well as the introduction of the Diplomate of National Board program with training conducted in district hospitals [35]. As a result, professional development opportunities, a supportive work environment, community integration, and work-life balance were factors that encouraged CHWs to choose rural areas [36].

Once I complete the rural posting then there is an opportunity for further professional growth and other career intentions.

[Doctor SM, 4-month interview]

#### Promote Local Food and Lifestyle

Access to local food and a lifestyle that aligns with their cultural values and traditions contribute to higher retention rates [37]. The availability of fresh, familiar foods and a slower pace of life compared to urban centers created a more appealing working environment for Indigenous CHWs.

When I eat food outside of my region, I face digestion problems. It may be because I am not used to spices and tastemakers. Our tribal food is simple and organic resulting in better health outcomes. Therefore, I prefer to be in rural areas.

[Nurse PK, 6-month interview]

#### Role of Cultural Beliefs and Practices

The study of sociocultural and economic factors that affected food consumption patterns in Arab countries demonstrates that the cultural beliefs and practices related to food significantly shaped dietary habits and food choices among rural communities [38]. However, in this study, CHWs reported that the ancient practices have a great impact and were driven by a need for local cuisine [39].

### Correlations Between Nutritional Support and Job Satisfaction

#### Better Health and Productivity

Access to nutritional support ensures that health care workers in rural areas stay physically fit and energized, which enhances their job performance [40]. Knowing that their health and well-being will be supported through nutritious, locally sourced food can make rural postings more attractive.

I have observed that rural people generally don't suffer from chronic diseases, but rather face issues like accidents, sunburn, sunstroke, or water-borne diseases. We are fortunate to have access to nutritious and healthy food.

[Nurse SH, 4-month interview]

#### Incentives of Fresh, Organic, and Local Food

Rural areas offer access to fresh, organic, and culturally significant local food. The availability of healthy, farm-to-table meals can serve as a strong motivator for health care workers, making rural postings more appealing due to the unique lifestyle benefits they offer.

They don’t pay me that time for the treatment I provide when I visit or am called to see patients. They often can’t afford to pay, but they give me fresh vegetables, pulses, or fruits that they harvest on the spot. Where else, in urban areas, can you find such genuine incentives and fresh produce?

[Nurse RJ, 6-month interview]

#### Low Cost of Living

In rural areas, access to fresh, local food can be more affordable than in urban settings. The prospect of spending less on quality food while still enjoying a nutritious diet can make rural postings more financially appealing.

I go to a market with 1000 INR [US $15] and buy groceries for the next two to three weeks. Everything is so cheap and fresh in the village markets. Do you think the same in the cities?

[Lab technician AG, 4-month interview]

#### Ethnicity

The findings demonstrated that ethnicity substantially impacted the food habits of a person owing to traditions, social norms, migration, and acculturation, which is evident within and outside India [41]. When one travels outside of their home country or region, this becomes quite apparent.

## Discussion

### Principal Findings

The study findings underscore the positive impact that rural alimentation plays in enhancing the contentment of CHWs and highlight the complex interplay between the rural work environment and the factors that drive their motivation [42]. The results indicate that CHWs with access to nutritious food experienced higher motivation and retention rates [43]. The objective of the study also aligns with previous research showing that psychological factors related to adopting a healthy diet can significantly boost life satisfaction and job motivation. In this study, CHWs expressed satisfaction and a sense of contentment with the availability and quality of food in rural areas. This is similar to the study conducted in Tanzania, which showed that access to nutritious food made CHWs more likely to remain in their positions for extended periods [44].

### Role of Nutrition in Enhancing Job Satisfaction

Previous studies have determined that a healthy diet helps protect against many chronic diseases, reducing the risk of developing such conditions [45,46]. The availability of locally sourced, nourishing food enhances rural health care workers’ motivation and urges providers and administrators to promote a local and healthy diet, which is a relatively simple and cost-effective strategy to improve CHW motivation and retention [47]. The impact of organic food remains to be determined; it helps reduce food safety risks such as pesticide residue and excessive additives [48].

While there is a strong correlation between nutrition and job satisfaction, few studies, especially in health-related fields, have explored this link. The job satisfaction and food habits of CHWs are largely influenced by their socioeconomic conditions and social and cultural practices. For Indigenous CHWs, local food products play a crucial role in maintaining their health and job satisfaction, which significantly impact their retention [49]. A balanced diet contributes to sustained energy and reduces feelings of fatigue and burnout, allowing workers to perform effectively, which enhances their satisfaction with their jobs. A study on nutra-ergonomics explores the relationship between workers, their work environment, and job satisfaction in connection to their nutritional status. It highlights nutrition as a key component of a safe and productive workplace, influencing physical and mental health, and contributing to long-term retention in their current roles [50].

### Cultural and Community Ties

Indigenous peoples typically share a deep ancestral connection to their lands and natural resources. They possess distinct cultures, languages, beliefs, and knowledge systems and maintain strong bonds with their land, properties, and territories. Their unique heritage and traditions are central to their identity and way of life. Culturally and politically, they will find themselves out of place from the rest of society [48].

### Impact of Nutritional Support

Nutrition contributes to many indicators of well-being, including maternal health, birth weight, child development, and oral health, and is an important determinant of chronic disease, which reduces life expectancy [51]. Inadequate nutritional intake is a major factor contributing to the burden of disease, and when individuals develop chronic conditions as a result, it often leads to significant out-of-pocket expenses for treatment [52,53].

### Government Policy

To attract and retain health workers in rural areas, both the state and central governments have implemented several monetary and nonmonetary benefits:

- Monetary incentives: (1) Hard area allowances and provision of residential facilities; (2) flexible salary schemes, such as the "You Quote, We Pay" strategy, ensuring competitive compensation; and (3) performance-based increments of up to 50% [35,54,55]
- Nonmonetary benefits: (1) Professional development opportunities for doctors, nurses, and allied health workers, including upskilling programs; (2) educational incentives, such as additional National Eligibility cum Entrance Test (Postgraduate) marks—10% for each year of service in remote or difficult areas, up to a maximum of 30%; (3) special honorariums to encourage rural practice among specialists; and (4) reservation of 50% of medical diploma seats for in-service state government doctors who have served in remote or challenging areas

These policies address financial and professional needs, making rural health care roles more attractive and sustainable [35,54]

### Implications of the Study

The study revealed several significant implications for the retention and motivation of CHWs in rural settings. It underscored that CHWs with access to nutritious and diverse local food products demonstrated higher motivation and retention rates.

First, enhancing the nutrition of CHWs leads to improved health outcomes within the communities they serve. Given their pivotal role in delivering primary health care services in resource-limited rural areas, ensuring the health and motivation of CHWs directly correlated with the quality of care provided to their communities. Second, addressing the nutritional requirements of CHWs assisted in mitigating the challenge of high turnover rates prevalent in rural areas. CHWs often encounter numerous obstacles that contribute to burnout and turnover, such as long working hours, inadequate remuneration, and inadequate support. Third, the study underscored the significance of tackling social determinants of health, including access to nutritious food, to enhance health care outcomes in underserved communities. By addressing these determinants, health disparities can be reduced, thereby fostering overall community health improvement.

### Limitations of the Study

The study was conducted in a specific geographic area and focused on a particular group of CHWs. The study’s lack of robust statistical representation may affect the reliability and generalizability of the results.

### Conclusion

The research investigated the relationship between rural alimentation and the motivation of Indigenous CHWs in Jharkhand, India. The findings demonstrated that the retention rates of Indigenous health care workers are positively influenced by their local cuisines and nutrition. Moreover, CHWs with access to organic and locally sourced food exhibited superior retention rates compared to Indigenous CHWs deployed in urban areas. This study also indicated that individuals often exhibit loyalty to their culinary preferences and dietary habits, which drives them to opt for local assignments. Consequently, rural sustenance plays a pivotal role in CHW retention, thereby enhancing the health outcomes of rural residents. In essence, the study underscored the significance of addressing the local diet requirements of CHWs to bolster their motivation and retention rates, consequently elevating the standard of health care services in rural settings. The implications drawn from the study hold crucial insights for policy makers and health care practitioners operating in similar contexts, offering valuable strategies for enhancing the retention and motivation of CHWs in rural areas.

## Supplementary material

10.2196/48346 Multimedia Appendix 1 Interview guidelines and questionnaire.

## Abbreviations

CHC: Community Health Centre
CHW: community health worker
