# Supplementary material for: The Impact of Rural Alimentation on the Motivation and Retention of Indigenous Community Health Workers in India: A Qualitative Study
Source: JMIRx Med. 2025 Jan 23;6:e48346. doi: 10.2196/48346 (PMC11781239; doi:10.2196/48346)
Supplement: Multimedia Appendix 1 [file xmed-v6-e48346-s001.docx]

**Interview Protocol Matrix**

| Dear Health worker,  I'm Ajit Kerketta, a research scholar at CHRIST UNIVERSITY Bengaluru, pursuing my PhD in healthcare management (HRM).  The study focuses on interviewing Indigenous Community Healthcare Workers who have spent more than five years working in rural areas and to my knowledge you fulfill the mentioned criteria. The title of my research is **" Exploring the Impact of Rural Alimentation on the Motivation and Retention of indigenous community health workers in Jharkhand, India: A Qualitative Study."** I'm conducting these interviews to learn about factors encouraging you to stay in Jharkhand's rural and remote health centres.  I would like to express my gratitude for consenting to participate in the interview portion of my research. As a result, the study aims to uncover the reasons that promote you to work in rural areas.  *During our informal discussion, you read the consent form indicating that I have permission (or not) to audio record our conversation*. *Are you still okay with me recording (or not) our conversation today? --- Yes ----No*  *If Yes: Thank you! Please let me know at any point if you want me to turn off the recorder or keep something you said off the record.*  *If No: Thank you for letting me know. I will take only notes of our conversation.*  *Prior to the interview, have you got any questions to be discussed?*  *If any questions arise at any point in this study, you can feel free to ask them at any time. I would be more than happy to answer your questions.* | |
| --- | --- |
| Name |  |
| Age |  |
| Qualification |  |
| Expertise |  |
| Designation |  |
| Name of current working place |  |
| Duration of stay in the current workplace |  |
| Profile/ CV | Yes/No |
| Phone No. |  |
| Ethical repayment |  |

Signature: ------ Date: ------- Time: -------

| **" Exploring the Impact of Rural Alimentation on the Motivation and Retention of Indigenous Community Healthcare Workers in Jharkhand, India:**  **A Qualitative Study"** | |
| --- | --- |
| **Semi-structure interview questions** | |
| 1 | Could you introduce yourself?   - Your family background. - Your childhood academic advancement - Geographical settings of your Residence? |
| 2 | Can you describe your role as a community health worker? |
| 3 | How long have you been working here, and what motivates you to continue? |
| 4 | What are the challenges you face as a community health worker? |
| 5 | What are your thoughts on food products in rural areas? |
| 6 | How do you view the relationship between nutrition and motivation? Do you believe there is a connection between proper nutrition and job satisfaction in retaining healthcare workers, or do you think there is no link? |
| 7 | Are health workers with better nutrition more motivated and likely to remain in their positions? Please explain. |
| 8 | How can the government contribute to promoting local food cultivation? |
| 9 | Please provide recommendations on how to attract more health workers to rural areas. |

Ajit Kerketta (1980152)

PhD Management Scholar

Christ Deemed to be University.

Bangalore, India

Mob. No. 8867055438

Email: [ajit.kerketta@res.christuniversity.in](mailto:ajit.kerketta@res.christuniversity.in)
